# Baseline microbiome and metabolome are associated with response to ITIS diet in an exploratory trial in patients with rheumatoid arthritis

**DOI:** 10.1002/ctm2.959

**Published:** 2022-07-08

**Authors:** Roxana Coras, Cameron Martino, Julia M. Gauglitz, Francesca Cedola, Anupriya Tripathi, Alan K. Jarmusch, Maram Alharthi, Marta Fernandez‐Bustamante, Meritxell Agustin‐Perez, Abha Singh, Soo‐In Choi, Tania Rivera, Katherine Nguyen, Tatyana Shekhtman, Tiffany Holt, Susan Lee, Shahrokh Golshan, Pieter C. Dorrestein, Rob Knight, Monica Guma

**Affiliations:** ^1^ Department of Medicine, School of Medicine University of California San Diego San Diego California USA; ^2^ Department of Medicine Autonomous University of Barcelona Barcelona Spain; ^3^ Center for Microbiome Innovation, Joan and Irwin Jacobs School of Engineering University of California San Diego San Diego California USA; ^4^ Department of Pediatrics, School of Medicine University of California San Diego San Diego California USA; ^5^ Bioinformatics and Systems Biology Program University of California San Diego San Diego California USA; ^6^ Department of Pharmacology Skaggs School of Pharmacy and Pharmaceutical Sciences University of California San Diego San Diego California USA; ^7^ Collaborative Mass Spectrometry Innovation Center University of California San Diego San Diego California USA; ^8^ Graduate Program in Biological Sciences, School of Biological Sciences, UCSD Division of Biological Sciences University of California San Diego San Diego California USA; ^9^ Department of Psychiatry University of California San Diego San Diego California USA; ^10^ Department of Computer Science and Engineering University of California San Diego San Diego California USA; ^11^ Department of Bioengineering University of California San Diego San Diego California USA; ^12^ Department of Medicine VA San Diego Healthcare System San Diego California USA

Dear Editor,

Changes in diet might modify the faecal microbiome and metabolomic profile, affecting pain in rheumatoid arthritis (RA). We examined the effect of an anti‐inflammatory “ITIS” diet^1^ on clinical outcomes, gut microbiome, and metabolome in RA patients, and found that baseline faecal microbiome and metabolome composition were associated with the pain response.

A prospective, open‐label pilot trial was conducted to evaluate a 2‐week isocaloric ITIS diet (Figure 1A) in patients with active RA. The study was approved by the Institutional Board Review. Change in pain (assessed on a visual analogue scale from 0 to 10) was the primary outcome. Patients were classified as responders (*N* = 7) or non‐responders (*N* = 13), based on the achievement of a 50% improvement in pain. Amplicon sequencing was used for microbiome profiling and untargeted metabolomics for metabolite analysis. Additional methods are included in the supporting information.

**FIGURE 1:**
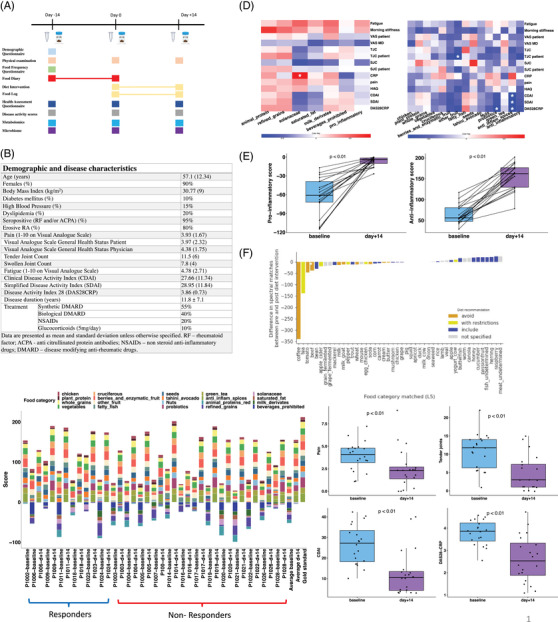
Trial design and baseline characteristics of patients, clinical and diet scores and adherence. (A) trial design; (B) Demographic and clinical characteristics of patients at recruitment; (C) Barplot representing the diet scores for each patient at baseline and after the ITIS diet intervention; the last bars show the average for all patients before and after the diet, as well as the gold standard score according to the proposed anti‐inflammatory diet; negative values correspond to consumption of pro‐inflammatory foods, prohibited in the proposed diet; the first seven pairs of barplots correspond to responders, while the rest correspond to non‐responders. (D) Heatmap representing partial correlation (Spearman) between pro‐inflammatory food groups (columns) and clinical (rows) scores. The significant correlations after correction for multiple hypothesis testing are signalled with a white asterisk; (E) Heatmap representing partial correlation (Spearman) between anti‐inflammatory food groups (columns) and clinical (rows) scores. The significant correlations after correction for multiple hypothesis testing are signalled with a white asterisk. Improvement in anti‐ and pro‐inflammatory diet scores after diet; paired samples Wilcoxon test was used to compare the means of the scores before and after the diet; (F) Differences in spectral matches between pre and post‐diet intervention. The difference after/before diet is shown, hence the negative bars represent a decrease in consumption, while positive bars are equivalent to an increase in consumption; (G) Average of clinical scores DAS28CRP, CDAI and pain before and after diet; paired samples Wilcoxon test was used to compare the means of the scores before and after the diet. RF – Rheumatoid factor; ACPA – anti‐citrullinated protein antibodies; CDAI – Clinical Disease Activity Index; SDAI – Simplified Disease Activity Index; DAS28 – Disease Activity Score using the 28‐joint count; SJC – swollen joint count; TJC – tender joint count; VAS MD – Visual Analogue Scale General Health Status Physician; PUFA ‐polyunsaturated fatty acids; MUFA – monounsaturated fatty acids

Twenty patients finalized the trial. Demographics and disease characteristics are summarized in Figure 1B. A diet score (212 = gold‐standard) was designed to characterize patients’ baseline diet (Figure 1C and Tables S1 and 2). Patients with higher baseline disease activity had lower anti‐inflammatory food scores, specifically fruit, probiotics, and anti‐inflammatory spices (Figure 1D).

Dietary intervention was well tolerated, and overall adherence based on the self‐reported diaries was approximately 70%, except for plant protein and probiotics, with final average scores less than 60% of the gold standard (Figure 1C,E and Table S3). We also assessed adherence using a reference data‐driven metabolomics approach. In large, the foods recommended increased while forbidden foods decreased post‐intervention (https://assets.researchsquare.com/files/rs‐654519/v1/bc74ec0e‐1d08‐4c67‐ad53‐98a73e03e3ff.pdf?c=1631886103 and Figure 1F).

Outcomes significantly improved post‐2‐weeks of the ITIS diet (Figure 1G and Tables S4 and 5). Pain improved from 3.89 ± 1.9 before versus 2.45 ± 2.4 after diet, *p* < .01 (Figure 1G). No significant change in BMI was observed (Figure S1A). Although obese patients (BMI ≥ 30) had higher disease activity, outcomes scores decreased in all patients (Figure S1B,C). There were no significant BMI changes in responders and non‐responders (Figure S1D). Baseline pain was similar in both groups (Figure S1E). Yet, patients that reached remission had lower DAS28CRP (Figure S1F).

Total diet scores before and after intervention were not different between responders and

non‐responders (Figure S2A). Yet, responders had a higher baseline anti‐inflammatory score than non‐responders (Figure S2B). Responders also had a less negative proinflammatory score than non‐responders after diet (meaning responders ate less forbidden ingredients than non‐responders) (Figure S2C). Additionally, patients with a higher baseline intake of whole grains, berries, enzymatic fruits, and unsaturated fat responded better to diet (Figure S2D–F). Yet, these scores were similar in responders and non‐responders post‐diet.

We next evaluated changes in microbiome and metabolome post‐intervention. Faecal microbiome and plasma and faecal metabolome alpha‐diversity didn't change over time (Figure 2A–C and Figure S3B). Changes in microbiome trajectories were very discrete, while they were more pronounced in both faecal and plasma metabolome (Figure S4). Different microbial and metabolic features increased or decreased post‐diet (Figure 2D–F). Interestingly, some metabolites are microbial metabolism products, including phenylacetylglutamine, bile acids (BA), and tryptophan/kynurenine.

**FIGURE 2:**
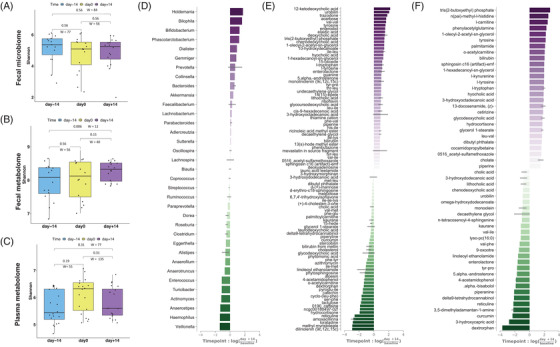
The Effect of the ITIS Diet on the Metabolome and Microbiome. (A–C) Comparison of average Shannon diversity of the microbiome (A), faecal metabolome (B) or plasma metabolome (C) across three timepoints; paired samples Wilcoxon tests were performed to compare means of the Shannon diversity between the different timepoints. Differential log‐fold ranking of microbes and metabolites by time for microbiome (D), faecal (E) or plasma metabolome (F). Positive values in the rank plot correspond to an increase on day+14, suggesting an increase after diet. Features presented in each plot are selected by the top and bottom‐ranked quartiles

We also evaluated if baseline microbiome or metabolome were associated with response. Baseline microbiome but not metabolome alpha‐diversity was significantly higher in responders (Figure 3A–C and Figure S3A), possibly reflecting the baseline dietary differences between the two groups (Figure S2D–F). Diet explains over 25% of the microbial structural variations in humans,^2^ and our data suggest that response to diet might be dependent on prior diet and microbiome. The microbiome and faecal metabolome beta‐diversity also showed differences between responders and non‐responders (Figure 3D–F), likely reflecting the baseline differences in diet and gut microbiome.

**FIGURE 3:**
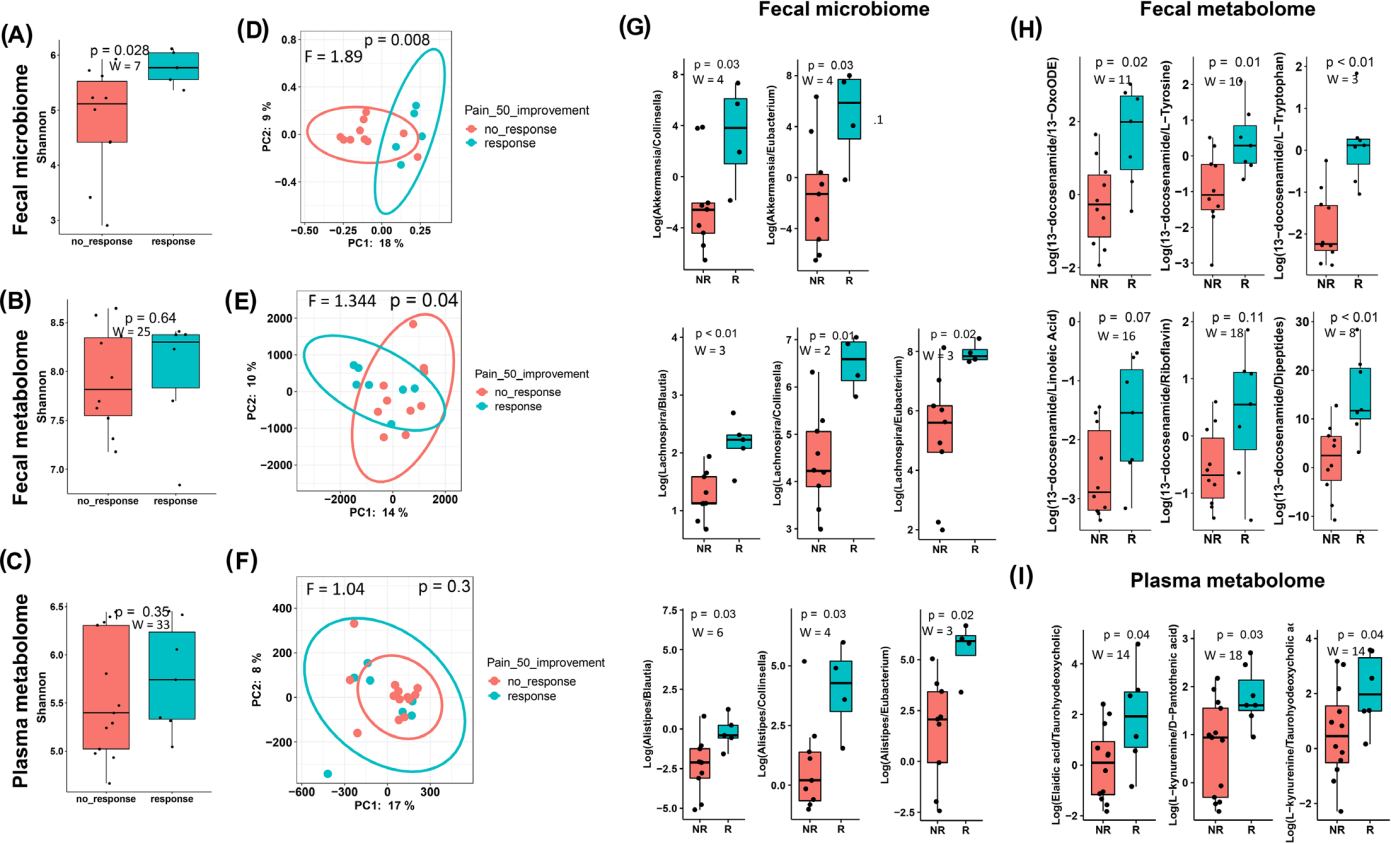
Diet scores and baseline microbiome and metabolome characteristics are associated with pain response to the ITIS diet. (A–C) Comparison of baseline Shannon‐diversity of the microbiome (A), faecal (B), or plasma metabolome (C) stratifying patients by the response. Mann‐Whitney U test was used to compare baseline Shannon diversity. (D) Baseline PCoA of Unweighted UniFrac of microbiome stratifying patients by the response. PERMANOVA was performed to compare beta‐diversity distances. (E, F) Baseline PCoA of Canberra distances of faecal (E) or plasma metabolome (F) stratifying patients by the response. PERMANOVA was performed to compare beta‐diversity distances. Comparison of differentially abundant ratios of microbes (G), faecal (H) and plasma (I) metabolomic features at baseline in responders (R) compared to non‐responders (NR); the ratios were obtained after performing songbird differential ranking analysis and visualization with Qurro; PCoA, principal coordinates analysis; non‐parametric tests (Mann‐Whitney U test were used to perform the comparison, the test statistic (W) and *p*‐value are presented for each comparison. 13‐oxoODE – 13‐keto‐9Z,11E‐octadecadienoic acid

Baseline log ratios of gut microbes and metabolites, as well as plasma metabolites, were different in responders and non‐responders (Figure 3G–I), suggesting their potential as predictive biomarkers of response to the ITIS diet. The log ratios *Akkermansia* to *Collinsella/Eubacterium*, *Lachnospira* to *Blautia/Collinsella/Eubacterium* and *Alistipes* to *Blautia/Collinsella/Eubacterium* were higher in R than NR. *Akkermansia* breaks down mucins converting them into short‐chain fatty acids, with anti‐inflammatory properties, whereas *Blautia* and *Dorea* were associated with other inflammatory diseases. These specific microbes might also be reflecting the baseline diet since *Lachnospira* was associated with vegetable intake.^3^

We further evaluated the relationship between the microbiomes and metabolomes changes post‐intervention with the pain response. Microbiome and metabolome alpha‐diversity did not change post‐ITIS diet in both responders and non‐responders (Figure 4A–C and Figure S3C). Gut microbiota is unique to each individual and relatively stable throughout life, yet it could be due to the short intervention of the current trial. Whether prolonged dietary changes can induce permanent alterations in the gut microbiota is unknown (review).^4^ Plasma metabolome beta‐diversity was different at day+14 between responders vs non‐responders (Figure 4D–F), suggesting that several circulating metabolites might be associated with pain post‐diet. Importantly, we identified microbiome changes at the genus level (Figure 4G). Since overall adherence was similar between responders and non‐responders, either the baseline microbiome of responders and/or some of the species that changed post‐intervention in responders metabolized the new ingredients and shifted the plasma metabolome to a new pool of circulating anti‐inflammatory metabolites. Anti‐inflammatory metabolites^5^, ^6^, ^7^ such as BA, l‐carnitine and acetyl‐carnitine, kynurenine, increased more in responders than non‐responders post‐diet (Figure 4G–I). Moreover, another BA, deoxycholic acid, co‐occurred with *Akkermansia* and was associated with response to pain (Figure 4J,K). Since BA and tryptophan are gut microbial products,^8^ microbiome differences throughout the trial seem to be associated with pain response in RA patients. Bile acids have been described to be involved in the regulation of immune cells^9^ and our findings require further studies to characterize their role in RA. Finally, we also detected differences in faecal and plasma abundances of some drugs that may reflect the effect of gut microbiome variations on their bioavailability.^10^

**FIGURE 4:**
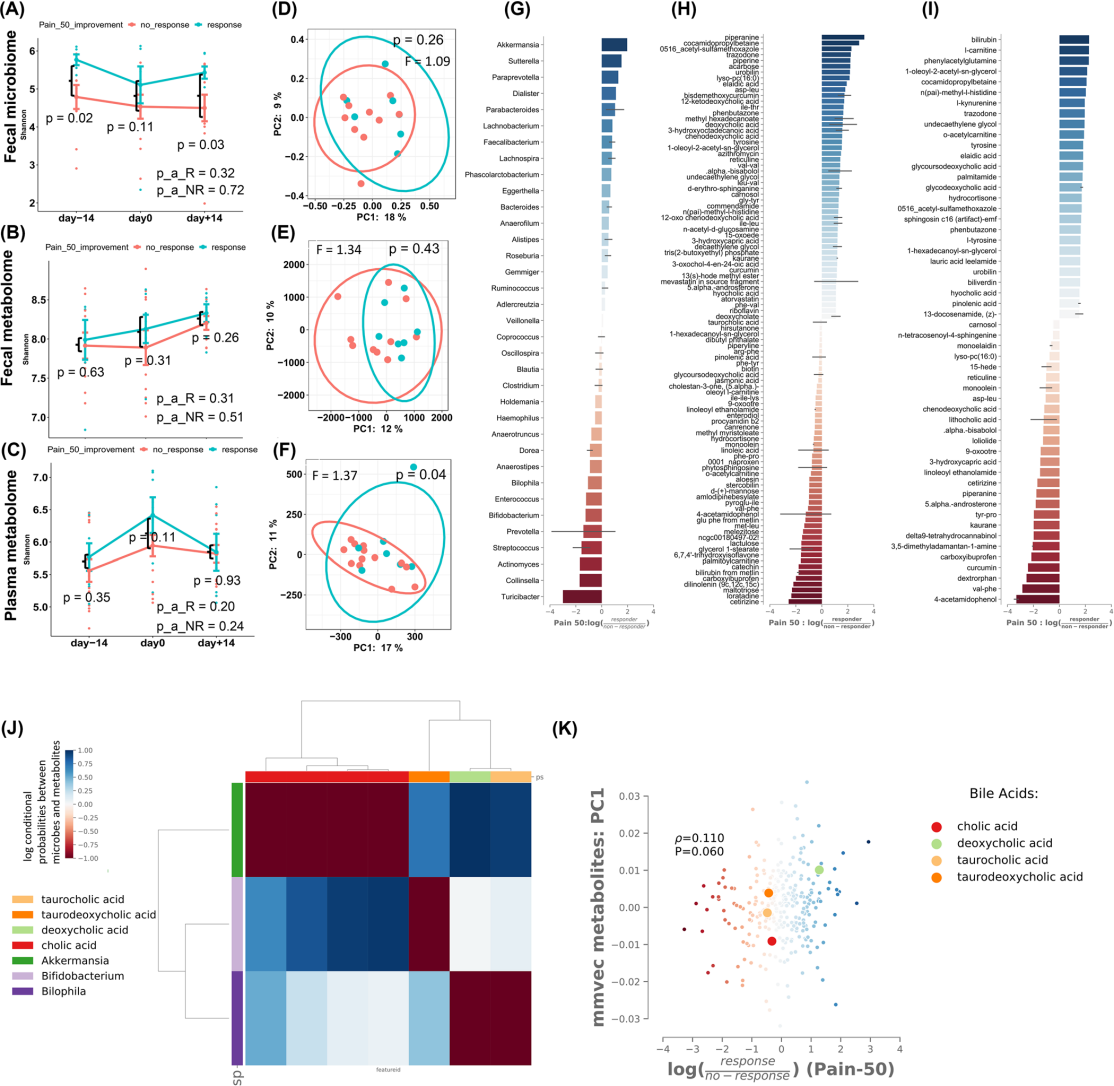
Changes in microbiome and metabolome characteristics are associated with pain response to the ITIS diet. (A–C) Changes in Shannon diversity of the microbiome (A), faecal (B) or plasma metabolome (C) according to Pain50 over the three timepoints. Mann‐Whitney U test was used to compare Shannon‐diversity between responders and non‐responders, and repeated measures ANOVA was used for the analysis of longitudinal data; (D) After diet PCoA of Unweighted UniFrac of microbiome according to pain50. (E, F) After diet PCoA of Canberra distances of faecal (E) or plasma (F) metabolome according to pain50. PERMANOVA was performed to compare beta‐diversity distances. (G–I) Songbird differential ranking of microbes (G), faecal metabolites (H) and plasma metabolites (I) Change in microbes and metabolites in responders compared to non‐responders. Positive values in the rank plot correspond to a positive increase of the features in responders compared to non‐responders. Features presented in each plot are selected by the top and bottom‐ranked quartiles. (J) Co‐occurrence analysis of microbes and faecal metabolome features. The plot represents the correlation coefficient between bile acids (in columns) and microbes (rows). The red colour indicates a negative correlation, while the blue colour indicates a positive correlation. (K) The co‐occurrence of microbes‐metabolites is influenced by Pain50 response to diet. The scatter plot represents the correlations between metabolites and microbiome features and the analysis is stratified by pain response: the blue dots represent correlations in responders, while the red dots represent correlations in non‐responders. The four bile acids that are emphasized represent correlations with metabolites in responders

To our knowledge, this is the first study to describe metabolomic and microbiome changes in RA after diet intervention. Several limitations including the number of patients, the lack of a control group, the short dietary intervention, and the lack of metagenomics to better understand the faecal and plasma metabolome, warrant further studies. In addition, metabolic tracing experiments in animal models will demonstrate the transfer of microbial (supporting information) anti‐inflammatory metabolites to the blood.

## CONFLICT OF INTEREST

All the authors declare no conflict of interest.

## FUNDING INFORMATION

Roxana Coras and Monica Guma were supported by the Krupp Endowed Fund. Roxana Coras was also supported by a UCSD Rheumatic Diseases Research Training Grant from the NIH/NIAMS (T32AR064194).

## Supporting information



Supporting information

Figure S1. Improvement in clinical scores is independent of changes in BMI
Figure S2. Diet scores and the relation with the pain response
Figure S3. Different microbiome alpha‐diversity indexes (faith, evenness and observed features) in relation to pain response
Figure S4. Trajectories of the microbiome (A), faecal (B) or plasma metabolome (C) between the timepoints for each patient
Table S2. Baseline diet scores
Table S3. Change in diet scores after diet
Table S4. Clinical outcomes across the three timepoints
Table S5. Number of responder/non‐responder patients by different outcomes
Table S6. Summary of dietary recommendations
Table S7. Proposed meal organization for the 2 weeks of the intervention
Table S8. Feasibility outcomes of the trial
Table S9. Demographic and clinical characteristics of R and NR

Table S1. Diet score calculation
Table S2. Baseline diet scores
Table S3. Change in diet scores after diet
Table S4. Clinical outcomes across the three timepoints
Table S5. Number of responder/non‐responder patients by different outcomes
Table S6. Summary of dietary recommendations
Table S7. Proposed meal organization for the 2 weeks of the intervention
Table S8. Feasibility outcomes of the trial
Table S9. Demographic and clinical characteristics of responders and non‐responders

## References

[1] Bustamante MF , Agustin‐Perez M , Cedola F , et al. Design of an anti‐inflammatory diet (ITIS diet) for patients with rheumatoid arthritis. Contemp Clin Trials Commun. 2020;17:100524. 32025586 10.1016/j.conctc.2020.100524 PMC6997513

[2] Rothschild D , Weissbrod O , Barkan E , et al. Environment dominates over host genetics in shaping human gut microbiota. Nature. 2018;555(7695):210‐215. 29489753 10.1038/nature25973

[3] Smith‐Brown P , Morrison M , Krause L , Davies PS . Dairy and plant based food intakes are associated with altered faecal microbiota in 2 to 3 year old Australian children. Sci Rep. 2016;6:32385. 27694811 10.1038/srep32385 PMC5046176

[4] Leeming ER , Johnson AJ , Spector TD , Le Roy CI . Effect of diet on the gut microbiota: rethinking intervention duration. Nutrients. 2019;11(12):2862. 10.3390/nu11122862 PMC6950569 31766592

[5] Haghighatdoost F , Jabbari M , Hariri M . The effect of L‐carnitine on inflammatory mediators: a systematic review and meta‐analysis of randomized clinical trials. Eur J Clin Pharmacol. 2019;75(8):1037‐1046. 30915521 10.1007/s00228-019-02666-5

[6] Wang S , Xu J , Zheng J , et al. Anti‐inflammatory and antioxidant effects of acetyl‐L‐carnitine on atherosclerotic rats. Med Sci Monit. 2020;26:e920250. 31945029 10.12659/MSM.920250 PMC6984015

[7] Haroon E , Welle JR , Woolwine BJ , et al. Associations among peripheral and central kynurenine pathway metabolites and inflammation in depression. Neuropsychopharmacology. 2020;45(6):998‐1007. 31940661 10.1038/s41386-020-0607-1 PMC7162907

[8] Agus A , Planchais J , Sokol H . Gut microbiota regulation of tryptophan metabolism in health and disease. Cell Host Microbe. 2018;23(6):716‐724. 29902437 10.1016/j.chom.2018.05.003

[9] Paik D , Yao L , Zhang Y , et al. Human gut bacteria produce TH17‐modulating bile acid metabolites. Nature. 2022;603(7903):907‐912. 10.1038/s41586-022-04480-z PMC9132548 35296854

[10] Scher JU , Nayak RR , Ubeda C , Turnbaugh PJ , Abramson SB . Pharmacomicrobiomics in inflammatory arthritis: gut microbiome as modulator of therapeutic response. Nat Rev Rheumatol. 2020;16(5):282‐292. 32157196 10.1038/s41584-020-0395-3 PMC11221369

