# Supplementary figures and images for: Baseline microbiome and metabolome are associated with response to ITIS diet in an exploratory trial in patients with rheumatoid arthritis
Source: Clin Transl Med. 2022 Jul 8;12(7):e959. doi: 10.1002/ctm2.959 (PMC9269999; doi:10.1002/ctm2.959)

Supplementary Figure 1

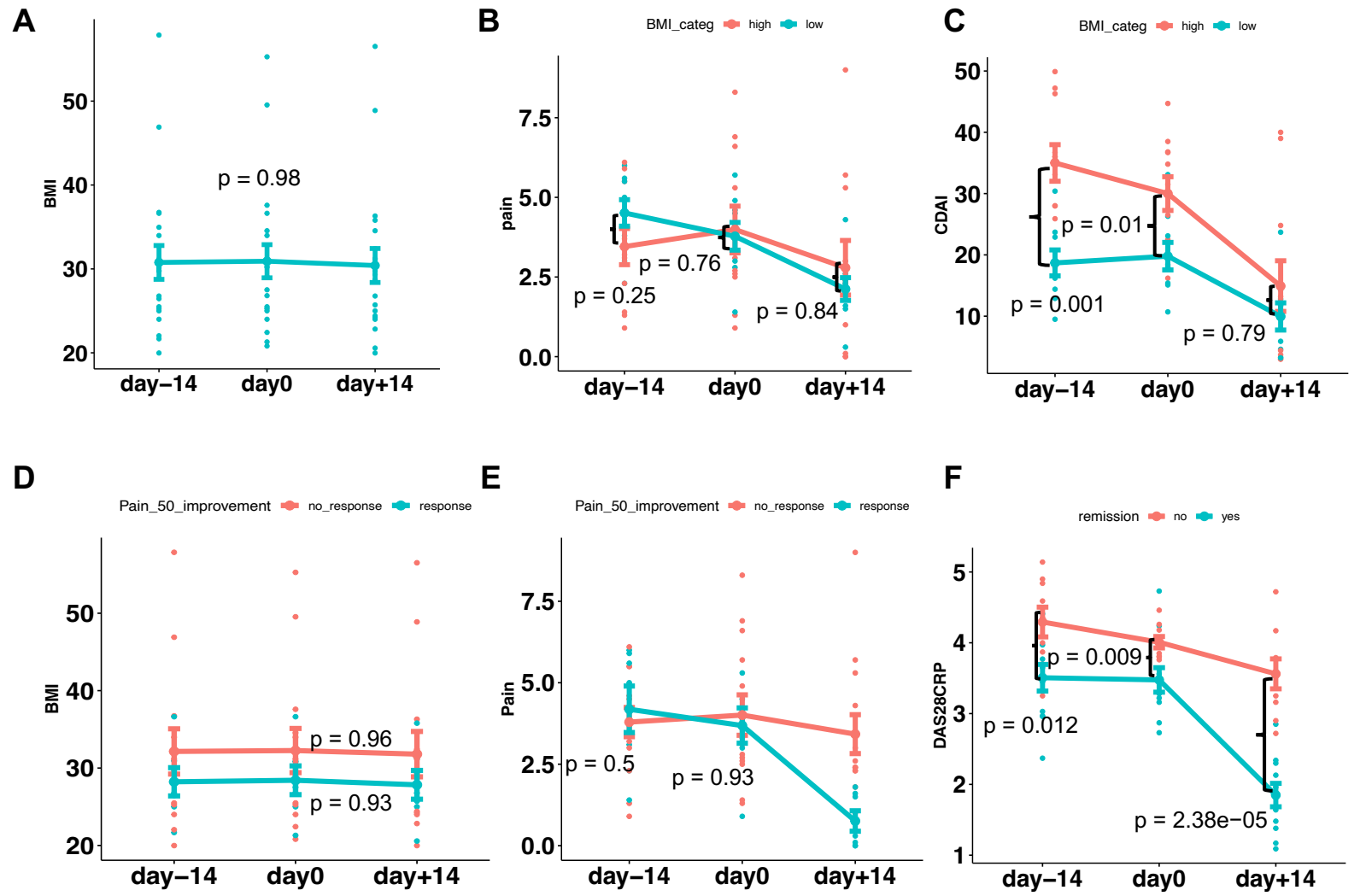

# Supplementary Figure 2

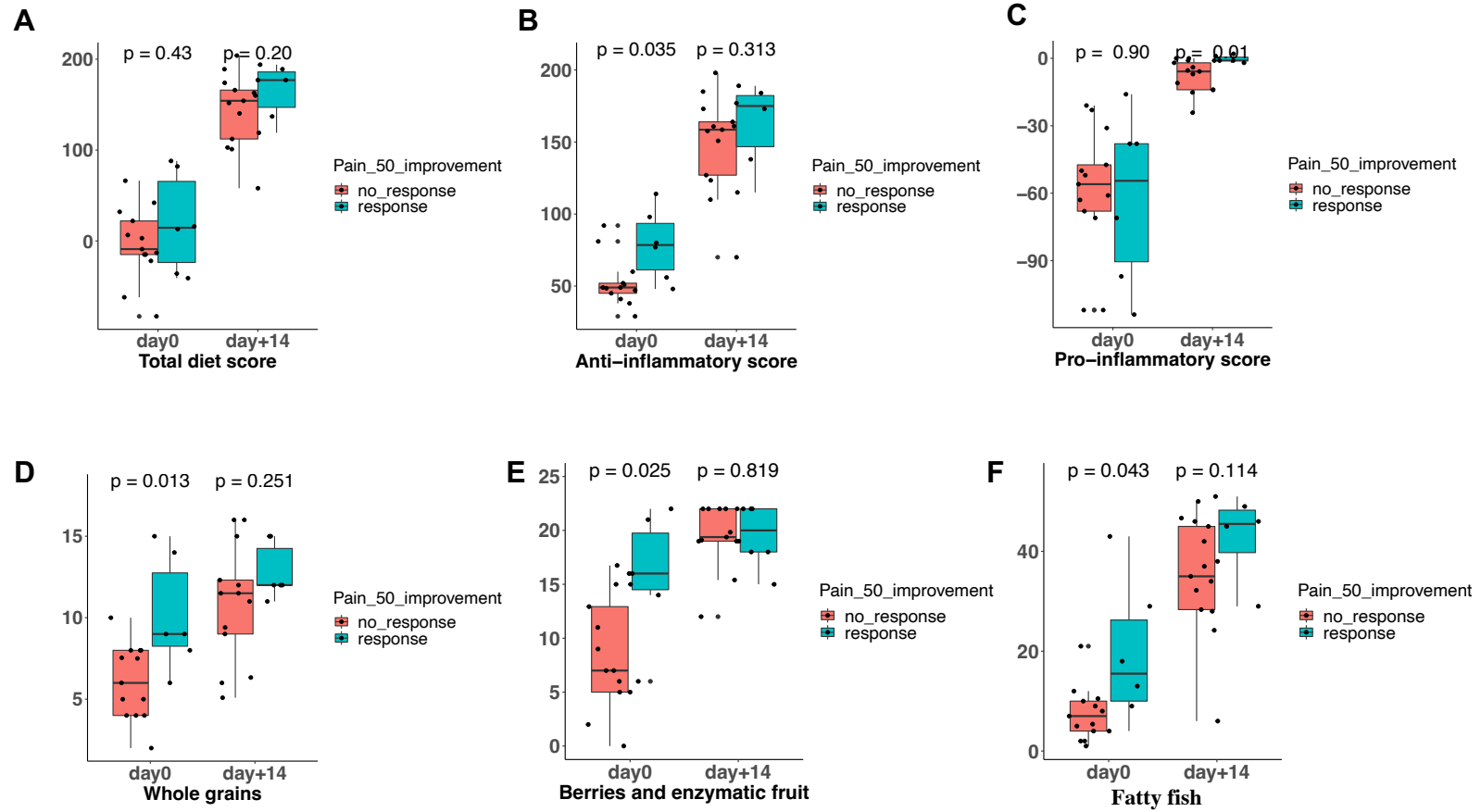

Supplementary Figure 3

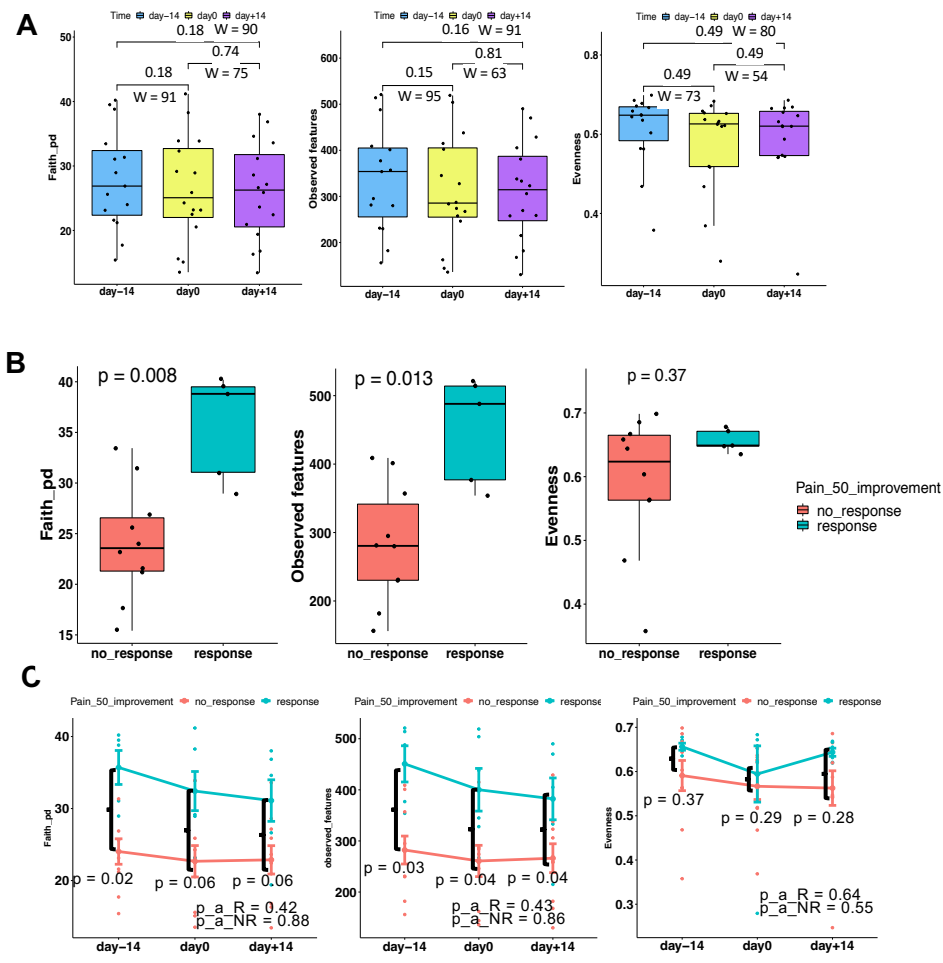

# Supplementary Figure 4

A

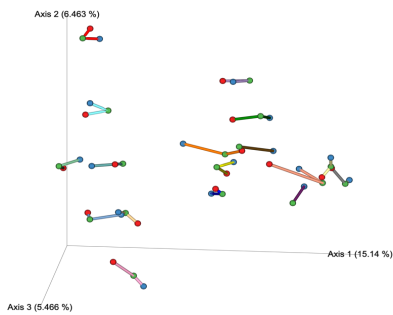

B

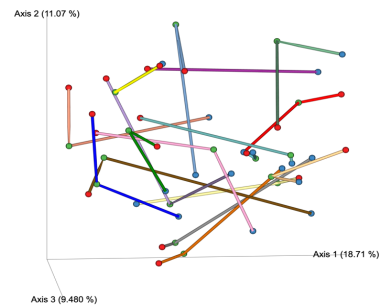

C

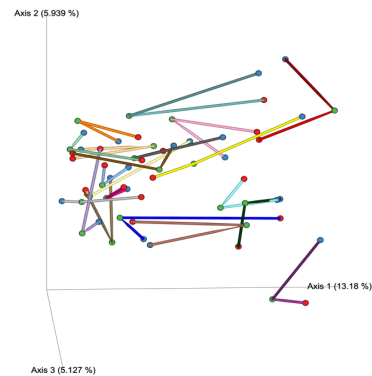

Supplement: Supplementary file 1 — Supporting information [file CTM2-12-e959-s001.pdf]
