# Supplementary material for: Baseline microbiome and metabolome are associated with response to ITIS diet in an exploratory trial in patients with rheumatoid arthritis
Source: Clin Transl Med. 2022 Jul 8;12(7):e959. doi: 10.1002/ctm2.959 (PMC9269999; doi:10.1002/ctm2.959)
Supplement: Supplementary file 2 — Figure S1. Improvement in clinical scores is independent of changes in BMI Figure S2. Diet scores and the relation with the pain response Figure S3. Different microbiome alpha‐diversity indexes (faith, evenness and observed features) in relation to pain response Figure S4. Trajectories of the microbiome (A), faecal (B) or plasma metabolome (C) between the timepoints for each patient Table S2. Baseline diet scores Table S3. Change in diet scores after diet Table S4. Clinical outcomes across the three timepoints Table S5. Number of responder/non‐responder patients by different outcomes Table S6. Summary of dietary recommendations Table S7. Proposed meal organization for the 2 weeks of the intervention Table S8. Feasibility outcomes of the trial Table S9. Demographic and clinical characteristics of R and NR [file CTM2-12-e959-s002.docx]

**Baseline Microbiome and Metabolome are Associated with Response to ITIS Diet in an Exploratory Trial in Patients with Rheumatoid Arthritis**

Roxana Coras^1,2^, Cameron Martino^3,4,5,#^, Julia M. Gauglitz^6,7,#^, Francesca Cedola^1^, Anupriya Tripathi^4,6,8^, Alan K. Jarmusch^3,4^, Maram Alharthi^1^, Marta Fernandez-Bustamante^1^, Meritxell Agustin-Perez^1^, Abha Singh^1^, Soo-In Choi^1^, Tania Rivera^1^, Katherine Nguyen^1^, Tatyana Shekhtman^9^, Tiffany Holt^9^, Susan Lee^1^, Shahrokh Golshan^9^, Pieter C. Dorrestein^3,4,6,7*^, Rob Knight^3,4,10,11*^, Monica Guma^1,2,12, *^

**SUPPLEMENTARY INFORMATION**

**Study Participants and Study Design:** A prospective, open label pilot trial was conducted to evaluate clinical and biological outcomes of a 2-week isocaloric ITIS diet^1^, with an average of ~1600 kcal/day, with the following distribution of the macronutrients: 22% protein, 45% carbohydrates and 29% fat. Approximately 77% of calories are plant based, while the rest come from animal products: 92% of carbohydrates, 83% of the fats and 44% of the protein are calories from plant products.^1^

Patients aged 18 or more with a diagnosis of RA defined by the 2010 ACR/EULAR classification criteria, with active disease (at least 3 swollen and 3 tender joints), and with no change in their standard of care treatment for the last 3 months, were recruited at Rheumatology Outpatient Clinic of the University California San Diego. The study was approved by the Institutional Board Review and patients signed an informed consent. Patients who were pregnant or lactating, already following a vegetarian or vegan diet, known food allergies, or patients who needed a change in RA medication regimen during the length of the study (30 days (D)) were excluded. We did not change their previous rheumatic treatments.

The study was performed from September 2018 to September 2019. Research visits were performed on 3 occasions during the study (Figure 1A). During their first visit (D-14), we established their clinical and biological baseline, and patients were asked to complete a daily diet log for 2 weeks before starting the diet. On their second visit (D0), two weeks later, patients were given instructions on how to follow the energy-adjusted ITIS diet (Table S6,7) and were also asked to complete a daily diet log for 2 weeks after starting the diet. On their third visit (D14), we evaluated diet adherence, trial satisfaction, and clinical parameters. At each visit, a thorough clinical examination was performed, which included the number of tender (TJC) and swollen joints ^2^. Pain and fatigue were evaluated by the patient, using a Visual Analogue Scale (VAS) that ranged from 0 to 10. HAQ (Health Assessment Questionnaire) was collected, and general health status evaluated by the patient (VAS_pt) and by the physician (VAS_MD) were also collected. Composite scores including Simplified Disease Activity Index (SDAI), Clinical Disease Activity Index (CDAI) and Disease Activity Index 28 (DAS28CRP) were calculated.

Blood samples were also collected at each visit by research personnel into 10 ml BD Vacutainer Blood Collection Tubes containing EDTA. Tubes were centrifuged for 20 min at 2000×rpm and plasma were transferred into 1.7ml tubes and immediately frozen and stored at −80 °C until analysis. We collected blood at all visits. Patients were asked to collect stool samples at home after each visit in special sealed plastic containers provided on the day of the visit. Stool samples were shipped in cooled containers, aliquoted and kept at -80 °C until analysis.

Thirty-nine patients were screened, twenty-six patients were recruited, and 20 patients went through the complete trial. Table S8 shows feasibility outcomes of the trial. The demographics of the patients, along with the disease characteristics are summarized in Figure 1B. The average age of RA patients was 57.1 years old (± 12.4) and 90% were females, with an average body mass index (BMI) of 30.7 (± 9). The average disease duration in years was 11.8 (± 7.1). The average number of tender joints (TJC) was 11.5 (± 6) and of swollen joints ^2^ 7.8 (± 4), with an average of 3.9 (± 1.67) of pain. The average CRP was 1.09 (± 0.98), while the mean DAS28CRP was 3.86 (± 0.73). Eleven patients were receiving treatment with combined synthetic disease modifying anti-rheumatic drugs (DMARDs) and 40% (8 patients) with biological DMARDs (3 patients Adalimumab, 1 Rituximab, 2 Abatacept, 1 Golimumab, and 1 Etanercept); amongst these patients, only 20% were receiving combined therapy with biological and synthetic DMARDs. Only 10% of patients received glucocorticoids (5mg/day), while 20% were receiving non-steroid anti-inflammatory drugs.

**Adherence to Dietary Intervention and Diet Score.** The subjects completed a Food Frequency Questionnaire (FFQ) on D-14. They also completed a daily record 2 weeks before and during the diet intervention, recording whether they had consumed the suggested meals and ingredients, which options they had chosen for breakfast and meals, and if there was any consumption of forbidden ingredients. We calculated a diet score (Table S1,2) for the evaluation of patient adherence. Consumption of anti-inflammatory foods according to the proposed diet50 received a positive evaluation. Pro-inflammatory foods, i.e., red meat or Solanaceae vegetables received a negative evaluation. We considered this score as the gold standard and used it to characterize the patient’s baseline diet as well. One patient did not fill in the dietary log between day -14 and day 0.

**Primary and Secondary Outcomes:** The primary endpoint was change in pain (assessed on a visual analogue scale from 0 to 10). Patients were classified as responders (R) or non-responders ^3^, based on achievement of a 50% improvement in pain after 2 weeks of following the diet. We considered as baseline the average of the outcome scores on day-14 and day 0, hence, to stratify patients we evaluated the outcomes on day+14 to baseline. Table S9 shows demographics and clinical characteristics of R and NR. Secondary outcomes included change of other clinical scores, body mass index (BMI), gut microbiota, fecal and plasma metabolome.

**Statistical analysis of Outcomes.** Continuous variables are presented as the mean ± standard deviation (SD), whereas categorical variables are summarized as the number (percentage) of subjects. Shapiro-Wilk test was used to assess normality of the data. Mann Whitney U test was used to compare 2 independent groups (i.e. clinical outcomes, diet scores and alpha diversity metrics between responders and non-responders at the same time-point, and in paired samples Wilcoxon test was used when 2 timepoints were compared (baseline versus day +14 for diet scores, clinical scores, and alpha diversity for microbiome and metabolites). ). Repeated measures ANOVA was used for the analysis of longitudinal data when 3 timepoints were compared. The barplots were built using the ggboxplot function and the line plots using the ggline function in the ggupbr package. The heatmaps of the correlations between clinical outcomes and diet scores were built using the heatmap.2 function, based on the partial Spearman correlation coefficients and corresponding P values. The partial Spearman correlation was performed using the ppcor package (https://cran.r-project.org/web/packages/ppcor/index.html) and the confounders included were BMI, hypertension, diabetes mellitus, dyslipidemia, duration of disease, treatment with corticosteroids, non-steroids anti-inflammatory drugs, synthetic disease modifying anti-rheumatic drugs and biological drugs). Correction for multiple hypothesis testing (Benjamini-Hochberg) was then performed. Data were analyzed with R 4.0.3 (https://www.r-project.org/).

**Untargeted Metabolomics**

**Sample preparation: Plasma**

Three hundred µL of MeOH (100%) was added to each well of the 96‐well plate Phree Phospholipid Removal Kit and centrifuged at 500 g for 5 minutes, 3 times prior to sample addition; the MeOH was discarded in the laboratory hazardous waste. Blood plasma was stored at −80°C prior to extraction in 1.5 mL microtubes. The blood plasma microtubes were thawed at room temperature prior to extraction. Blood plasma samples were placed into one of four Phree Phospholipid Removal Kit 96‐well plates randomly. The thawed blood plasma samples were vortexed for 5 seconds and centrifuged for 1 minute at 5,000 rpm prior to pipetting 50 µL of each sample into the 96‐well Phree Phospholipid Removal Kit. Two hundred µL of MeOH (100%) was added to each well using a multichannel pipette; the solution was aspirated and dispensed 5 times to mix the blood plasma and organic solvent. A 96‐well plate (Eppendorf Microplate 96/U‐PP) was placed under the Phree Phospholipid Removal Kit to collect the sample and centrifuged at 500 g for 5 minutes. The Phree Phospholipid Removal Kit portion was discarded in the solid biohazardous waste and the sample‐containing 96‐well plate was evaporated until dry using a CentriVap Benchtop Vacuum Concentrator (Labconco, Kansas City, MO). The 96‐well plate containing the dried extract was covered (Storage Mat III 3080) and stored at −80°C prior to analysis. Immediately prior to analysis, the dried extract material was resuspended in 200 µL of MeOH‐water (1:1), sonicated for 5 minutes, centrifuged for 5 minutes at 500 g, and covered with a plate‐sealing film (Zone‐Free Sealing Films).

**Sample preparation: Feces**

Fecal samples were stored at −80°C prior to extraction. The swab tip of the BD Falcon SWUBE Collection and Transport System swabs were cut into Nunc 96‐Well Polypropylene DeepWell Storage Plates. Sample barcodes were scanned using a barcode scanner and saved into a Google Sheets spreadsheet generating a record of which sample was positioned in each well of the plate. Three hundred µL of MeOH‐water (1:1) was added to each well using a multichannel pipette. The deep well plate was covered with a storage mat and floated in an ultrasonic bath for 5 minutes. The samples were placed in a 4°C refrigerator overnight to extract. Subsequently, the swabs were removed from each well using tweezers, rinsing in between with nanopure water. The swab tips were disposed of in the solid biohazardous waste. Extracts were evaporated until dry using a CentriVap Benchtop Vacuum Concentrator (Labconco, Kansas City, MO, USA). The 96‐well plates containing the dried extract were covered (96‐deep well plate mats, Nunc 96 Well Caps for 1.0 mL Polystyrene DeepWell Plates) and stored at −80°C prior to analysis. Immediately prior to analysis, the dried extract material was resuspended in 300 µL of MeOH‐water (1:1), sonicated for 5 minutes, and centrifuged for 5 minutes at 500 g. One hundred µL of extract from each well was transferred into a 96‐well plate (Eppendorf Microplate 96/U‐PP) and diluted twofold using MeOH‐water (1:1) and covered with a plate‐sealing film (Zone‐Free Sealing Films).

**Data acquisition:** Blood plasma and fecal samples were analyzed using LC-MS/MS data acquisition which was performed on a Vanquish ultrahigh-performance liquid chromatography (UPLC) system using a core-shell silica C18 column (2·1 x 50 mm, 1·7-μm particle size, 100-Å pore size; Kinetex, Phenomenex) coupled to a Q Exactive Orbitrap mass spectrometer (Thermo Fisher Scientific, Bremen, Germany). Five microliters of sample were injected and run at 0.5 ml/min on a gradient of solvent A (HPLC-grade water with 0·1% formic acid) and solvent B (HPLC-grade acetonitrile with 0.1% formic acid). The column was maintained at 40 °C. The UPLC elution gradient ran for 12·5 min per sample: 5% B from 0 min to 1 min, a linear gradient of 5 to 100% B over 8 min, a hold at 100% B for 2 min, a return to 5% B over 0·5 min, and a hold at 5% B for 2 min to equilibrate the column for the next sample. The flow was directed into a heated electrospray ionization source operated in positive ionization mode with the following parameters: an auxiliary gas flow rate of 14 arbitrary units (a.u.), sweep gas flow rate of 3 a.u., sheath gas flow rate of 52 a.u., spray voltage of +3·5 kV, capillary temperature of 270°C, auxiliary gas heater temperature of 435°C, and S-Lens RF level of 50. The data-dependent acquisition mode was used to acquire the data in which MS1 scans from *m/z* 100 to 1,500 (scan rate, 7 Hz) were followed by an MS2 scan, specifically a product ion scan produced using stepped normalized collision energy higher-energy collisional dissociation, of the five most abundant ions from the prior MS1 scan.

**Data processing and data analysis:** Raw data were uploaded to MassIVE (https://massive.ucsd.edu/), converted to .mzML files, imported into MZmine2 ^4^, and truncated at *m/z* 1500 and a 9.5-min retention time (RT). The parameters used in MZmine2 are as follows. Mass detection was performed with a noise threshold of 2·0e5 for MS1 and 2·0e3 for MS2 in centroid mode, and chromatograms were built with a 0·05-min time span, 1·0e6 minimum height, and 10-ppm m/z tolerance. Chromatograms were deconvoluted with the baseline cutoff algorithm and a minimum peak height of 1·0e6, peak duration of 0·05 to 1·0 min, and baseline of 1.0e4. Isotope peak removal was performed with 15-ppm *m/z* tolerance, 0.3 min RT tolerance, and maximum charge of 4, and peaks were aligned with 10-ppm *m/z* tolerance, 75 weight *m/z* tolerance, and 0·4 min RT tolerance. Gap filling was also performed with 10% intensity tolerance, 15-ppm *m/z* tolerance, and 0·3 min RT tolerance. Peaks were also filtered to remove singletons found in only one sample. Both MS1 and MS2 feature tables were exported, and the “export for GNPS'' feature was used to generate a .mgf file for GNPS. The signal intensities of the MS1 features were normalized (probabilistic quotient normalization) to the sulfamethoxazine internal standard.

**Molecular networking (GNPS):** A molecular network was created with the feature based molecular networking workflow (https://ccms‐ucsd.github.io/GNPSDocumentation/featurebasedmolecularnetworking/) on GNPS (<https://gnps.ucsd.edu/ProteoSAFe/status.jsp?task=8a1c9d53f2db45728d82033e734e583f>)^5^. The data were filtered by removing all MS2 product ions within ± 17 *m/z* of the precursor *m/z*. MS2 spectra were window filtered by choosing only the top 6 fragment ions in the ± 50 *m/z* window throughout the spectrum. The precursor *m/z* tolerance was set to 0·02 *m/z* and a MS2 product ion m/z tolerance of 0·02 *m/z*. A network was then created where edges were filtered to have a cosine score above 0·7 and at least 4 matched peaks. Further, edges between two nodes were kept in the network if and only if each of the nodes appeared in each other’s respective top 10 most similar nodes. Finally, the maximum size of a molecular family (i.e., network component) was set to 100, and the lowest scoring edges were removed from molecular families until the molecular family size was below this threshold. The spectra in the network were then searched against GNPS spectral libraries. The library spectra were filtered in the same manner as the input data. All matches kept between network spectra and library spectra were required to have a score above 0·7 and at least 4 matched peaks. The annotations are level 2 or 3 according to the 2007 metabolomics standards initiative^6^.

**Metabolomics data availability:** All MS data (.d and .mzXML files) are publically available via GNPS/MassIVE (massive.ucsd.edu), a public MS data repository, under the accession number MSV000084556. Differentially abundant features (metabolites) between pain 50 improvement and study D+14 and -14 were calculated through Songbird (see Differential abundance and multi-omics analyses). Posteriorly, only the annotated metabolites were used to generate the barplots presented in the figures. The comparisons for all the features are presented as supplementary tables.

**Microbiome**

**Sample and data processing:** 17 patients provided fecal samples, which were sequenced using the V4 region of the 16S rRNA gene. Fifteen samples with >5,000 reads were included in the final analysis. The total number of OTUs identified across all samples was *n* = 4739. The Earth Microbiome Project 28 DNA extraction and 16S rRNA gene sequencing protocol was used for sample processing. In brief, stool sample DNA was extracted from swabs using the 96‐well MoBio Powersoil DNA kit, and barcoded 515F‐806R primers targeting the V4 region of the 16S rRNA gene were used for 16S amplification. The resulting V4 amplicons were sequenced at UCSD Institute for Genomic Medicine on an Illumina MiSeq. Raw 16S rRNA gene sequencing data were uploaded to Qiita, where it was demultiplexed, trimmed to 150 bp reads, and processed to suboperational taxonomic units (sOTU)s using Deblur^7^. The resulting table contained 3,356 ASVs with an average total read count of 99,404 (standard deviation of 14,208 counts) and a minimum of 62,568 counts and maximum of 134,654 counts. The feature table and representative sequences were then downloaded and further analyzed with QIIME 2^8^ to perform, taxonomic assignments, phylogenetic tree generation, and differential abundance ranking, and to calculate a and b-diversity. Taxonomic assignments used the naive bayes sklearn classifier in QIIME 2 trained on the 515F/806R region of Greengenes^9^ 13_8 99% operational taxonomic units (OTUs). Phylogenetic tree generation was performed by inserting representative sequences into the Greengenes 13_8 99% tree with SEPP^10^ in QIIME 2. No rarefaction was done before performing a-diversity (observed OTUs) and b-diversity (unweighted UniFrac)^11^ calculations in QIIME 2. b-diversity statistical significance between sample groups was assessed through permutational multivariate analysis of variance (PERMANOVA) (Anderson MJ. 2017. Permutational multivariate analysis of variance (PERMANOVA), p 1–15. In Balakrishnan N, Colton T, Everitt B, Piegorsch W, Ruggeri F, Teugels JL (ed), Wiley StatsRef: statistics reference online. John Wiley & Sons, Ltd, Chichester, United Kingdom). Statistical significance of a-diversity comparisons between groups (responders and non-responders) was determined with Mann Whitney U test, while the comparison of the diversity between the 3 timepoints was performed using repeated measures ANOVA, with post hoc analysis and Benjamini-Hochberg correction for multiple hypothesis testing. Data visualizations were generated with the R packages phyloseq^12^, ggplot and ggpubr.

**Differential abundance and multi-omics analyses:** Differential abundance analysis of fecal microbiome, metabolomics, and plasma metabolomics was performed with Songbird, which is described in detail in the methods paper ^13^, to estimate differential rankings of features between sample groups (i.e., microbes or metabolites). Briefly, differential abundance methods rely on a normalization (in the model) rather than a transformation (performed on the data directly). The multinomial regression model in Songbird is formulated with an additive log-ratio transformation (alr) normalization, which is similar to ALDEx2^14^. Optimized model parameters were determined for a formula of pain 50 improvement, time, and the interaction of both terms through an exhaustive grid search compared by the Q-squared score (model coefficient of variation / pseudo-model coefficient of variation). Where a Q-squared value of greater than 0 represents an acceptable model fit. Q-squared values of 0·01, 0.2, and 0·03 were obtained for the fecal microbiome, metabolomics, and plasma metabolomics data, respectively. The resulting feature rankings were confirmed through two-sided Student’s t-test on log-ratios through Qurro^15^. Multi-omic analysis of fecal microbiome and metabolomics data were performed through mmvec (microbe-metabolite vectors), a neural network method for producing log-conditional probabilities of cooccurrence between microbial and metabolite features visualized as heat maps and paired latent representations in few dimensions, which can be visualized in scatterplot or biplot ordinations^16^. A high conditional probability of close spatial similarity in the ordination indicates high cooccurrence between a microbe and metabolite pair, while a negative conditional probability or a high spatial distance in the ordination indicates low cooccurrence. The mmvec model parameters were optimized (batch size of 4000, 50 epochs, and 6 latent dimensions) to minimize the low cross-validation error and model likelihood. Differential cooccurrence patterns (mmvec microbe-metabolite interactions) in relation to the exposure treatment were evaluated by correlating the mmvec PC1 loading with respect to the songbird log fold change differential with respect to each songbird fecal metabolomics differential. The only significant correlation was found with Pain 50 metabolites (Spearman rho=0·35; P=8·12 x 10^-8^).

**Microbiome data availability:** The 16S rRNA gene sequencing data are publicly available in Qiita ^17^ at<https://qiita.ucsd.edu/study/description/12766>. The code used for analysis can be accessed at: <https://github.com/roxanacoras/ITIS_diet>.

**Supplementary Figure 1. Improvement in Clinical Scores is Independent of Changes in BMI.** A) Evolution of BMI in all patients across the 3 visits. B) Evolution of CDAI in patients with a higher BMI (>=30, red) compared to patients with a lower BMI (< 30, blue); C) Evolution of pain in patients with a higher BMI (>=30, red) compared to patients with a lower BMI (< 30, blue); D) Comparison of BMI evolution in patients with a 50% improvement in pain (response, blue) compared with patients with a lower improvement in pain (no response, red); E) Evolution of pain score in patients who will present an improvement of 50% of pain (response – blue), compared to patients who will have a lower improvement in pain (no response – red); F) Comparison DAS28CRP evolution in patients who reach remission (yes, blue) compared to the ones who don’t (no, red); BMI – body mass index; CDAI – Clinical Disease Index; DAS28CRP – Disease Activity Score using the 28-joint count and C reactive protein. Mann Whitney U test was used to compare the means at each timepoint between responders and non-responders.

**Supplementary Figure 2. Diet scores and the relation with pain response.** A-C) Comparison of baseline and after diet total (A), anti-inflammatory (B), or pro-inflammatory (C) scores in patients, stratifying by response; D-F) Comparison of baseline and after diet consumption of whole grains (D), berries and enzymatic fruit (E), or unsaturated fats (F) consumption, stratifying by response.

**Supplementary Figure 3. Different microbiome alpha-diversity indexes (Faith, evenness and observed features) in relation to pain response.** A) Comparison of different microbiome alpha-diversity indexes across 3 timepoints B) Comparison of baseline microbiome alpha-diversity stratifying patients by response; C) Changes in microbiome alpha-diversity indexes stratifying the patients by pain50 over the 3 timepoints.

Supplementary Figure 4. Trajectories of microbiome (A), fecal (B) or plasma metabolome (C) between the timepoints for each patient.

**Supplementary Table 1. Diet score calculation.** The table shows the importance given to each type of food/ingredient in the calculation of the score. Positive scores were given to foods considered anti-inflammatory, while negative scores were assigned to foods considered pro-inflammatory

**Supplementary Table 2. Baseline diet scores.** The table depicts the average score assigned to each food group for patients before the D, as well as the gold standard score for the diet we propose. The food groups we defined as follows: animal protein (red meat, eggs), refined grains (biscuits, white bread, breakfast cereals - commercial), solanaceae (eggplant, tomato, potato), saturated fat (precooked and processed food, butter), milk derivates (milk and derivates, flavored yoghurt), beverages prohibited (alcohol, coffee, sweetened beverages – soda, energy drinks, fruit drinks), pr-inflammatory spices (sauces, added sugars – pastries, sugar), chicken (poultry), plant protein (legumes), whole grains (whole grains, pseudocereals, oats), vegetables (all vegetables except cruciferous, greens and non-greens), cruciferous (cauliflower, cabbage, kale, garden cress, bok choy, broccoli, Brussels sprouts), berries and enzymatic fruit (berries, enzymatic fruit), fruit (all the other fruit), fatty fish, seeds (flaxseeds oil, seeds), tahini_avocado (tahini or avocado), nuts (walnuts), probiotics (miso, plain yogurt), green tea (green tea), anti-inflammatory spices (black pepper, ginger, turmeric, cinnamon, vanilla).

**Supplementary Table 3. Change in diet scores after diet.**

**Supplementary Table 4. Clinical outcomes across the 3 timepoints.** The clinical outcomes at the 3 timepoints are presented, along with the pairwise comparisons p values.

**Supplementary Table 5. Number of responder/non responder patients by different outcomes.**

**Supplementary Table 6**. **Summary of Dietary Recommendations.** The table includes recommendations of foods recommended to increase intake and foods which are recommended to decrease intake.

**Supplementary Table 7. Proposed meal organization for the 2 weeks of the intervention.**

**Supplementary Table 8**. **Feasibility outcomes of the trial.** The table includes feasibility outcomes of the trial.

**Supplementary Table 9.** **Demographic and clinical characteristics of R and NR**. DMARD – disease modifying anti-rheumatic drugs; BMI – body mass index; NSAIDs – non steroid anti-inflammatory drugs; DM – diabetes mellitus; HTN – hypertension; HLD - hyperlipidemia

**REFERENCES**

1. Bustamante MF, Agustin-Perez M, Cedola F, et al. Design of an anti-inflammatory diet (ITIS diet) for patients with rheumatoid arthritis. *Contemp Clin Trials Commun.* 2020;17:100524.

2. Divangahi M, Aaby P, Khader SA, et al. Trained immunity, tolerance, priming and differentiation: distinct immunological processes. *Nat Immunol.* 2021;22(1):2-6.

3. Timilsina S, Brittan K, O'Dell JR, et al. Design and Rationale for the Veterans Affairs "Cooperative Study Program 594 Comparative Effectiveness in Gout: Allopurinol vs. Febuxostat" Trial. *Contemp Clin Trials.* 2018;68:102-108.

4. Pluskal T, Castillo S, Villar-Briones A, Oresic M. MZmine 2: modular framework for processing, visualizing, and analyzing mass spectrometry-based molecular profile data. *BMC Bioinformatics.* 2010;11:395.

5. Wang M, Carver JJ, Phelan VV, et al. Sharing and community curation of mass spectrometry data with Global Natural Products Social Molecular Networking. *Nat Biotechnol.* 2016;34(8):828-837.

6. Sumner LW, Amberg A, Barrett D, et al. Proposed minimum reporting standards for chemical analysis Chemical Analysis Working Group (CAWG) Metabolomics Standards Initiative (MSI). *Metabolomics.* 2007;3(3):211-221.

7. Amir A, McDonald D, Navas-Molina JA, et al. Deblur Rapidly Resolves Single-Nucleotide Community Sequence Patterns. *mSystems.* 2017;2(2).

8. Bolyen E, Rideout JR, Dillon MR, et al. Reproducible, interactive, scalable and extensible microbiome data science using QIIME 2. *Nat Biotechnol.* 2019;37(8):852-857.

9. McDonald D, Price MN, Goodrich J, et al. An improved Greengenes taxonomy with explicit ranks for ecological and evolutionary analyses of bacteria and archaea. *ISME J.* 2012;6(3):610-618.

10. Janssen S, McDonald D, Gonzalez A, et al. Phylogenetic Placement of Exact Amplicon Sequences Improves Associations with Clinical Information. *mSystems.* 2018;3(3).

11. Lozupone C, Knight R. UniFrac: a new phylogenetic method for comparing microbial communities. *Appl Environ Microbiol.* 2005;71(12):8228-8235.

12. McMurdie PJ, Holmes S. phyloseq: an R package for reproducible interactive analysis and graphics of microbiome census data. *PLoS One.* 2013;8(4):e61217.

13. Morton JT, Marotz C, Washburne A, et al. Establishing microbial composition measurement standards with reference frames. *Nat Commun.* 2019;10(1):2719.

14. Fernandes AD, Reid JN, Macklaim JM, McMurrough TA, Edgell DR, Gloor GB. Unifying the analysis of high-throughput sequencing datasets: characterizing RNA-seq, 16S rRNA gene sequencing and selective growth experiments by compositional data analysis. *Microbiome.* 2014;2:15.

15. Fedarko MW, Martino C, Morton JT, et al. Visualizing 'omic feature rankings and log-ratios using Qurro. *NAR Genom Bioinform.* 2020;2(2):lqaa023.

16. Morton JT, Aksenov AA, Nothias LF, et al. Learning representations of microbe-metabolite interactions. *Nat Methods.* 2019;16(12):1306-1314.

17. Gonzalez A, Navas-Molina JA, Kosciolek T, et al. Qiita: rapid, web-enabled microbiome meta-analysis. *Nat Methods.* 2018;15(10):796-798.
