# Supplementary material for: Baseline microbiome and metabolome are associated with response to ITIS diet in an exploratory trial in patients with rheumatoid arthritis
Source: Clin Transl Med. 2022 Jul 8;12(7):e959. doi: 10.1002/ctm2.959 (PMC9269999; doi:10.1002/ctm2.959)
Supplement: Supplementary file 3 — Table S1. Diet score calculation Table S2. Baseline diet scores Table S3. Change in diet scores after diet Table S4. Clinical outcomes across the three timepoints Table S5. Number of responder/non‐responder patients by different outcomes Table S6. Summary of dietary recommendations Table S7. Proposed meal organization for the 2 weeks of the intervention Table S8. Feasibility outcomes of the trial Table S9. Demographic and clinical characteristics of responders and non‐responders [file CTM2-12-e959-s003.docx]

**Supplementary Table 1. Diet score calculation**

| Food groups | Food category | Score | What to do if they eat more |
| --- | --- | --- | --- |
| Protein | Poultry (3 times a week) | 6 | Max 6 |
|  | Red meat | 0 | Subtract if > 0 |
|  | White or Fatty fish (3 times a week, per 2) | 12 | Max 12 |
|  | Seafood | 0 | Subtract if > 2 |
|  | Legumes (3 times a week) | 6 | Max 6 |
|  | Eggs | 0 | if > 4 /week - subtract |
|  | Total | **24** |  |
|  |  |  |  |
| Bread and cereals | Whole grains | 8 | Max 8 |
|  | Refined grains | 0 | Subtract if > 0 |
|  | Pseudocereals | 4 | Max 4 |
|  | Oats | 4 | Max 4 |
|  | Biscuits, white bread, other cereals | 0 | Subtract if > 0 |
|  | Total | **16** |  |
|  |  |  |  |
| Vegetables&mushrooms | Greens (includes lettuce, spinach, other types of green leafy vegetables, ) | 8 | Max 8 |
|  | Nongreens (celery, peppers, cucumber, artichoke, onion, radishes, carrot…) | 8 | Max 8 |
|  | Cruciferous (cauliflower, cabbage, kale, garden cress, bok choy, broccoli, Brussels sprouts) | 16 | Max 16 |
|  | Tomato | 0 | Subtract if > 0 |
|  | Potato | 0 | Subtract if > 0 |
|  | Eggplant | 0 | Subtract if > 0 |
|  | Total | **32** |  |
|  |  |  |  |
| Fruit | Berries | 8 | Max 8 |
|  | Other fruits | 14 | Max 14 |
|  | Enzymatic fruit | 14 | Max 14 |
|  | Total | **36** |  |
|  |  |  |  |
| Fat | Saturated (precooked, processed food, butter) | 0 | Subtract if > 0 |
|  | Polyunsaturated (flaxseeds oil, seeds) (daily) | 14 | Max 14 |
|  | Monounsaturated (tahini or avocado) (daily) | 14 | Max 14 |
|  | Total | **28** |  |
|  |  |  |  |
| Nuts | Walnuts | 14 | Max 14 |
|  | Total | **14** |  |
|  |  |  |  |
| Miso | Miso (4 times x 3) | 12 | Max 12 |
|  |  |  |  |
| Dairy | Milk and derivates | 0 | Subtract if > 0 |
|  | Plain Yogurt | 14 | Max 14 |
|  | Flavored yogurt | 0 | Subtract if > 0 |
|  | Total | **14** |  |
|  |  |  |  |
| Drinks | Coffee | 0 | Subtract if > 0 |
|  | Alcohol | 0 | Subtract if > 0 |
|  | Green tea (daily) | 14 | Max 14 |
|  | Sweetened beverages (Soda, energy drinks, fruit drinks) | 0 | Subtract if > 0 |
|  | Total | **14** |  |
|  |  |  |  |
| Added sugars | Sugar, pastries | **0** | Subtract if > 0 |
|  |  |  |  |
| Sauces/spices | Turmeric - Ginger- Black Pepper (14 per turmeric/black pepper/daily, 4 per ginger) | 18 | Max 18 |
|  | Cinnamon | 2 |  |
|  | Vanilla | 2 |  |
|  | Sauces | 0 | Subtract if > 0 |
|  | Total | **22** |  |
|  |  |  |  |
| Total |  | 212 |  |

| **Supplementary Table 2. Baseline diet scores** | | |
| --- | --- | --- |
| **Food** | Day 0 | Gold Standard |
| **Pro-inflammatory foods** | | |
| **Animal protein** | -8.32 (±6.77) | 0 |
| **Refined grains** | -12.96 (±8.78) | 0 |
| **Solanaceae** | -5.49 (±4.9) | 0 |
| **Saturated fat** | -4.14 (±3.65) | 0 |
| **Milk derivates** | -6.31 (±4.76) | 0 |
| **Beverages prohibited** | -14.79 (±9.19) | 0 |
| **Pro-inflammatory spices** | -10 (±8.48) | 0 |
| **Total pro inflammatory score** | -62.01 (±30.33) | 0 |
| **Anti-inflammatory** | | |
| **Chicken** | 4.11 (±2.1) | 6 |
| **Plant protein** | 3.19 (±2.44) | 6 |
| **Whole grains** | 7.37 (±3.28) | 16 |
| **Vegetables** | 10.18 (±2.48) | 16 |
| **Cruciferous** | 5.26 (±5.17) | 16 |
| **Berries and enzymatic fruit** | 10.88 (±6.27) | 22 |
| **Other fruit** | 8.01 (±4.33) | 14 |
| **Fatty fish** | 4.61 (±4.22) | 12 |
| **Seeds** | 2.48 (±3.95) | 14 |
| **Avocado and tahini** | 2.86 (±4.15) | 14 |
| **Nuts** | 1.25 (±2.25) | 14 |
| **Probiotics** | 1.33 (±1.76) | 26 |
| **Green tea** | 1.04 (±2.1) | 14 |
| **Anti-inflammatory spices** | 0.89 (±1.66) | 22 |
| **Total anti-inflammatory score** | 63.47 (±22.93) | 212 |
| **Total score** | 1.46 (±44.92) | 212 |

| **Supplementary Table 3. Change in diet scores after diet** | | | | |
| --- | --- | --- | --- | --- |
| **Food** | Day 0 | Day+14 | p value | Gold Standard |
| **Pro-inflammatory foods** | | | | |
| **Animal protein** | -8.32 (±6.77) | -1.14 (±2.11) | <0.0001 | 0 |
| **Refined grains** | -12.96 (±8.78) | -1.27 (±1.64) | < 0.001 | 0 |
| **Solanaceae** | -5.49 (±4.9) | -0.62 (±0.94) | < 0.001 | 0 |
| **Saturated fat** | -4.14 (±3.65) | -0.26 (±0.9) | < 0.001 | 0 |
| **Milk derivates** | -6.31 (±4.76) | -0.51 (±0.95) | < 0.001 | 0 |
| **Beverages prohibited** | -14.79 (±9.19) | -0.59 (±1.42) | < 0.001 | 0 |
| **Pro-inflammatory spices** | -10 (±8.48) | -2.39 (±2.71) | 0.001 | 0 |
| **Total pro inflammatory score** | -62.01 (±30.33) | -6.79 (±7.58) | < 0.001 | 0 |
| **Anti-inflammatory** | | | | |
| **Chicken** | 4.11 (±2.1) | 4.76 (±1.9) | 0.317 | 6 |
| **Plant protein** | 3.19 (±2.44) | 3.15 (±2.34) | 0.894 | 6 |
| **Whole grains** | 7.37 (±3.28) | 11.48 (±3.23) | 0.001 | 16 |
| **Vegetables** | 10.18 (±2.48) | 15.47 (±1.87) | < 0.001 | 16 |
| **Cruciferous** | 5.26 (±5.17) | 11.48 (±6.04) | 0.005 | 16 |
| **Berries and enzymatic fruit** | 10.88 (±6.27) | 19.46 (±2.88) | < 0.001 | 22 |
| **Other fruit** | 8.01 (±4.33) | 10.02 (±3.78) | 0.166 | 14 |
| **Blue fish** | 4.61 (±4.22) | 6.69 (±3.7) | 0.106 | 12 |
| **Seeds** | 2.48 (±3.95) | 13.04 (±2.81) | <0.001 | 14 |
| **Avocado and tahini** | 2.86 (±4.15) | 8.9 (±4.39) | 0.001 | 14 |
| **Nuts** | 1.25 (±2.25) | 8.85 (±4.06) | <0.001 | 14 |
| **Probiotics** | 1.33 (±1.76) | 15.21 (±7.06) | < 0.001 | 26 |
| **Green tea** | 1.04 (±2.1) | 9.99 (±5.09) | < 0.001 | 14 |
| **Anti-inflammatory spices** | 0.89 (±1.66) | 16.89 (±2.95) | < 0.001 | 22 |
| **Total anti-inflammatory score** | 63.47 (±22.93) | 155.4 (±31.3) | < 0.001 | 212 |
| **Total score** | 1.46 (±44.92) | 148.61 (±36.48) | < 0.001 | 212 |

| Supplementary Table 4. Clinical outcomes across the 3 timepoints | | | | | | |  |  |
| --- | --- | --- | --- | --- | --- | --- | --- | --- |
| Variable | | **Day-14** | **Day0** | **Day+14** | **Day-14 Day0** | **Day-14 Day +14** | **Day0**  **Day +14** | |
| Morning stiffness | | 40.75 (39.943) | 46.65 (50.202) | 29.25 (33.922) | 0.735 | 0.318 | 0.08 | |
| Fatigue | | 4.78 (2.719) | 3.975 (2.894) | 2.49 (2.387) | 0.329 | 0.001 | 0.029 | |
| VAS Patient | | 3.975 (2.328) | 3.28 (2.328) | 2.455 (2.401) | 0.323 | 0.008 | 0.207 | |
| VAS MD | | 4.385 (1.754) | 4.425 (1.723) | 1.975 (1.95) | 0.993 | <0.001 | <0.001 | |
| TJC | | 11.5 (5.996) | 10.15 (5.441) | 4.55 (4.861) | 0.59 | <0.001 | 0.001 | |
| TJC Patient | | 12.55 (8.593) | 10.7 (8.578) | 4.65 (5.092) | 0.599 | <0.001 | 0.008 | |
| SJC | | 7.8 (3.995) | 7.55 (2.8) | 3.7 (3.743) | 0.949 | <0.001 | <0.001 | |
| SJC Patient | | ﻿9.3 (7.974) | ﻿5.5 (5.226) | ﻿3.05 (3.98) | 0.047 | 0.001 | 0.264 | |
| CRP | | 1.29 (0.985) | 1.198 (1.277) | 0.921 (0.872) | 0.924 | 0.297 | 0.5 | |
| HAQ | | 0.8 (0.666) | 0.732 (0.666) | 0.565 (0.67) | 0.636 | 0.009 | 0.08 | |
| SDAI | | 28.951 (11.848) | 26.602 (8.97) | 13.601 (11.501) | 0.591 | <0.001 | <0.001 | |
| SDAI Patient | | 31.5 (17.396) | 25.102 (14.413) | 13.052 (10.699) | 0.122 | <0.001 | 0.001 | |
| DAS28CRP | | 3.86 (0.731) | 3.715 (0.521) | 2.619 (1.044) | 0.748 | <0.001 | <0.001 | |
| DAS28CRP Patient | | 3.929 (0.97) | 3.462 (1.021) | 2.525 (1.034) | 0.151 | <0.001 | 0.001 | |

| Data are presented as mean and standard deviation unless otherwise specified. BMI - body mass index; VAS - visual analogue scale; MD - medical doctor; TJC - tender joint count; SJC - swollen joint count; CRP - C reactive protein; HAQ - health assessment questionnaires; CDAI - clinical disease activity index; SDAI - simplified disease activity index; DAS28 - disease activity score that includes 28 joints |
| --- |

| Supplementary Table 5. Number of responder/non responder patients by different outcomes | | |
| --- | --- | --- |
| Response Criteria | **Responders** | **Non-Responders** |
| 50 % Pain improvement | 7 | 13 |
| 50% Fatigue improvement | 9 | 11 |
| 50% Morning Stiffness improvement | 12 | 6 |
| 50% VAS Patients improvement | 6 | 14 |
| 50% VAS Physician improvement | 14 | 6 |
| 50% HAQ improvement | 10 | 10 |
| 20% CDAI improvement | 16 | 4 |
| 50% CDAI improvement | 11 | 9 |
| 70% CDAI improvement | 8 | 12 |
| ACR20 | 11 | 9 |
| ACR50 | 6 | 14 |
| Remission (DAS28CRP) | 10 | 10 |
| Low disease activity (CDAI) | 8 | 12 |

VAS - visual analogue scale; HAQ - health assessment questionnaires; CDAI - clinical disease activity index; SDAI - simplified disease activity index; DAS28 - disease activity score that includes 28 joints

**Supplementary Table 6. Summary of dietary recommendations**

| **Main recommendations (WHAT)** | **Diet strategies (HOW)** |
| --- | --- |
| ·Increase omega 3 lipid intake and decrease omega 6 lipid intake (red meat, frying and refined oils, pre-cooked food) | ·The diet must contain oily fish such as two days in the week |
|  | Daily intake of avocado and or sesame seeds or tahini |
|  | Condiment with flaxseed oil |
| ·Increase the consumption of green leafy vegetables and fruits | ·Daily green leafy vegetables (arugula, lettuce, broccoli, zucchini, Green beans) |
|  | Daily home-made Green juice (made of fruits and green vegetables |
| ·Introduce the consume of prebiotics and probiotics | ·Daily yogurt or miso |
| ·To reinforce particular types of cooking | ·Recommendation of steam cooking, baking and not too long boiling, and decrease frying and long boiling processes |
| ·To introduce enzymatic fruits | ·Daily enzymatic fruit (pineapple, mango or papaya) as a snack |
| ·To eliminate sauces and introduce anti-inflammatory condiments | ·Condiment with turmeric and/or black pepper |
|  |  |
| ·To decrease solanaceae vegetables and introduce vegetables with anti-inflammatory properties | ·Forbidding the consumption of eggplant, tomatoes and potatoes and suggest consumption of garlic, onion, carrot, pumpkin, zucchini or others |
| ·To substitute red meat per other types of proteins | ·Reduce animal meat to chicken or turkey to twice a week |
|  | Two-three times a week, introduce legumes (red, white beans, lentils or garbanzo) |
|  | Two-three eggs per week |
|  | Two-three days per week, eat fish |
| ·To reduce the consumption of gluten | ·To reduce the intake of wheat bread and change it for rye bread or corn tortillas |
| ·To delete dairy products (except yogurt) due its content of large proteins | ·To forbid dairy products and to suggest consumption of vegetable milks (almond, rice, coconut) |
| ·To introduce chia seeds, since they contain high quantities of tryptophan, serotonin precursor associated with control of pain | ·Suggested for salads and/or juices |
| ·To maintain a good acidic-basic balance by introducing alkalinizing juices | ·Daily home-made Green juice (made of fruits and green vegetables) |

**Supplementary Table 7. Proposed meal organization for the 2 weeks of the intervention**

|  |  | **DAY 1 and 8** | | **DAY 2 and 9** | **DAY 3 and 10** | **DAY 4 and 11** | **DAY 5 and 12** | **DAY 6 and 13** | **DAY 7 and 14** |
| --- | --- | --- | --- | --- | --- | --- | --- | --- | --- |
| **7-8 am** | **SMOOTHIE** | Coconut milk, mango, papaya, pineapple | | Pear, lemon, yogurt, vanilla, water | Grapes, celery, spinach, cucumber, lime, water | Almond or oat milk, spinach, strawberries, pear, chia seeds, cinnamon and ginger | Spinach, ginger, turmeric, papaya, flaxseeds, banana, water | Papaya, spinach, almond milk, turmeric, chia seeds, honey | Parsley, pineapple, strawberries, water |
| **7-8 am** | **BREAKFAST** | 1-2 spoons of oats with non-dairy milk (oat milk, almond milk or rice milk). Add berries (optional). | | 1-2 corn tortillas, spread with avocado, sesame seeds, and flaxseed oil. | 1 -2 corn tortillas with tahini (sesame seed extract) with ¼ teaspoon of honey . | 1-2 spoons of oat with non-dairy milk (oat milk, almond milk or rice milk. Add berries (optional). | 1-2 corn tortillas, spread with avocado, sesame seeds, and flaxseed oil. | 1 -2 corn tortillas with tahini (sesame seed extract) with ¼ teaspoon of honey. | 1-2 corn tortillas, spread with avocado, sesame seeds, and linseed oil. |
|  |  | Green tea infusion. | | Green tea infusion. | Green tea infusion | Green tea infusion. | Green tea infusion. | Green tea infusion | Green tea infusion. |
| **10-11 am** | **SNACK** | Plain yogurt (Chobani Brand, no sugar added) | | Plain yogurt (Chobani Brand, no sugar added) | Plain yogurt (Chobani Brand, no sugar added) | Plain yogurt (Chobani Brand, no sugar added) | Plain yogurt (Chobani Brand, no sugar added) | Plain yogurt (Chobani Brand, no sugar added) | Plain yogurt (Chobani Brand, no sugar added) |
| **12-1 pm** | **LUNCH** |  | | | | | | | |
|  |  | OPTION 1: Salad (generous plate) | | | | | | | |
|  |  | OPTION 2: Grains with vegetables | | | | | | | |
|  |  | OPTION 3: Legumes with vegetables | | | | | | | |
| **4:00 PM** | **SNACK** | Mango, papaya, pineapple, apple, pear or banana + 4 walnuts | Mango, papaya, pineapple, apple, pear or banana + 4 walnuts | | Mango, papaya, pineapple, apple, pear or banana + 4 walnuts | Mango, papaya, pineapple, apple, pear or banana + 4 walnuts | Mango, papaya, pineapple, apple, pear or banana + 4 walnuts | Mango, papaya, pineapple, apple, pear or banana + 4 walnuts | Mango, papaya, pineapple, apple, pear or banana + 4 walnuts |
| **7-8 pm** | **DINNER** |  | | | | | | | |
|  |  | OPTION 1: Vegetable soup/cream + protein | | | | | | | |
|  |  | OPTION 2: Miso soup + baked/steamed/grilled vegetables + protein | | | | | | | |
|  |  | OPTION 3: Salad + protein | | | | | | | |

**Supplementary Table 8: Feasibility outcomes of the trial**

|  | Screening |
| --- | --- |
| Patients screened | 39 |
| Number excluded | 13 |
| Reasons for exclusion | Unable to contact - 3  Did not want to do the diet trial once they knew more - 7  Decided to start standard of care therapy - 3 |
| Biological DMARDs | Did not want to take more pills – 6  Did not want to escalate therapy because of side effects – 6  Believe that diet could help - 14 |
|  | **Enrollment** |
| Number consented and enrolled | 26 |
| Number withdrawn | 6 |
| Reasons for withdrawal | Lost contact – 1  Headache due to coffee withdrawal – 1  Moved – 1  Lack of time - 3 |
| Number completed the protocol | 20 |
|  | Adverse effects |
| Description | Headache due to coffee withdrawal - 1 |

**Supplementary Table 9. Demographic and clinical characteristics of responders and non-responders**

| Variable | Non-responders (N=13) | Responders  (N=7) | p |
| --- | --- | --- | --- |
| Steroids | 15.38% | 0 | 0.75 |
| DMARDs | 61.53% | 57.14% | 1 |
| NSAIDs | 23.07% | 14.28% | 1 |
| Biological DMARDs | 30.74% | 57.14% | 0.5 |
| Age | 55.07 (±13.69) | 62.14 (±7.47) | 0.15 |
| BMI | 32.22 (±11) | 28.22 (±4.83) | 0.29 |
| DM | 15.38% | 0 | 0.75 |
| HBP | 7.69% | 28.57 | 0.55 |
| DL | 23.07% | 14.28 | 1 |

DMARD – disease modifying anti-rheumatic drugs; BMI – body mass index; NSAIDs – non steroid anti-inflammatory drugs; DM – diabetes mellitus; HBP – high blood pressure; DL - dyslipidemia
